# Epitope screening and vaccine molecule design of PRRSV GP3 and GP5 protein based on immunoinformatics

**DOI:** 10.1111/jcmm.18103

**Published:** 2024-01-12

**Authors:** Dongyu Liu, Yaping Chen

**Affiliations:** ^1^ Heilongjiang Bayi Agricultural University Daqing China

**Keywords:** GP3, GP5, immunoinformatics, porcine reproductive and respiratory syndrome virus, vaccine candidate

## Abstract

Porcine reproductive and respiratory syndrome (PRRS) is a respiratory disease in pigs that causes severe economic losses. Currently, live PRRSV vaccines are commonly used but fail to prevent PRRS outbreaks and reinfection. Inactivated PRRSV vaccines have poor immunogenicity, making PRRSV a significant threat to swine health globally. Therefore, there is an urgent need to develop an effective PRRSV vaccine. This study used immunoinformatics to predict, screen, design and construct a candidate vaccine that fused B‐cell epitopes, CTL‐ and HTL‐dominant protective epitopes of PRRSV strain's GP3 and GP5 proteins. The study identified 12 B‐cell epitopes, 6 CTL epitopes and 5 HTL epitopes of GP3 and GP5 proteins. The candidate vaccine was constructed with 50S ribosomal protein L7/L1 molecular adjuvant, which has antigenicity, solubility, stability, non‐allergenicity and a high affinity for its target receptor, TLR‐3. The C‐ImmSim immunostimulation results showed significant increases in cellular and humoral responses (B cells and T cells) and production of TGF‐β, IL‐2, IL‐10, IFN‐γ and IL‐12. The constructed vaccine was stable and immunogenic, and it can effectively induce strong T‐cell and B‐cell immune responses against PRRSV. Therefore, it is a promising candidate vaccine for controlling and preventing PRRSV outbreaks.

## 1 INTRODUCTION

Porcine reproductive and respiratory syndrome (PRRS) is an acute infectious disease caused by the porcine reproductive and respiratory syndrome virus (PRRSV). The virus strain was first reported and isolated in China in 1996.^1^, ^2^ Outbreaks and epidemics of PRRSV infection, leading to mass mortality, have significantly threatened the pig farming industry worldwide.^3^, ^4^, ^5^, ^6^ With the prevalence and evolution of PRRSV, the clinical characteristics have changed from the original reproductive disorders to high pathogenic PRRS (HP‐PRRS) in pig herds.^7^ PRRSV is constantly mutated in nature, resulting in the continuous emergence of new virus strains, leading to the difficulty of its complete elimination, prevention and control.^8^, ^9^, ^10^, ^11^, ^12^, ^13^ In order to quickly and effectively prevent and control PRRSV, vaccine immunization is considered to be the best way. Commercial markets currently offer live attenuated vaccines; however, increasing controversy exists about their efficacy in providing complete protection.^14^ Therefore, developing a rapid, accurate, safe and effective PRRS vaccine is still particularly important during the current epidemic.

The PRRSV is a positive‐sense, single‐stranded RNA virus, which belongs to the arteritis virus.^15^ The genome of PRRSV is approximately 15 kb in size, the ORF1a and ORF1b encode non‐structural proteins including Nsp 1–12 proteins, and ORF2‐7 encodes structural proteins including GP2, GP3, GP4, GP5, M, E and N proteins.^16^ The main outer membrane protein of PRRSV is GP5 protein, which is the most mutable structural protein. The extracellular domain of GP5 is an important target for neutralizing antibodies.^17^ Its N‐glycosylation changes are related to the neutralization and pathogenicity of the virus.^18^ Also, the secondary outer membrane glycoprotein, GP3 protein, plays important roles in pathogenicity, replication, assembly and mutation.^19^ GP3 is the major structural and immunogenic protein of PRRSV, which stimulates both cellular and humoral immunity.^20^ In addition, studies have shown that constructing a fusion protein GP3–GP5 vaccine using reverse genetic technology can improve the protective immunity of pigs against PRRSV infection.^21^ However, the technology cannot effectively prevent and control the constantly mutating PRRSV. Recently, with the rapid development of high‐throughput sequencing technology, vaccine development using immunoinformatics has gradually become a frontier direction in life science research.^22^ The study employed immunoinformatics methods to screen the gene source and identify immunoprotective epitopes that could enhance the affinity of protective antibodies and promote a balanced cellular immunity. The construction of candidate vaccines can effectively induce neutralizing antibodies that inhibit virus replication, kill infected cells and balance the effector T cells of the immune response.^23^ Based on this, our team targeted the isolated PRRSV, GP3 and GP5 proteins and screened the dominant protective epitopes using immunoinformatics methods. Furthermore, we concatenated the dominant epitope of the fusion GP3–GP5 protein as a candidate vaccine, which provides a feasible new solution for developing and preparing the PRRSV vaccine.

## 2 MATERIALS AND METHODS

### 2.1 Prediction and screening of PRRSV GP3 and GP5 protein sequences and linear B‐cell epitopes

The GP3 and GP5 proteins in the HP‐PRRSV strains isolated by our laboratory were used as target proteins for screening multi‐epitope vaccines against HP‐PRRSV. Firstly, this study predicted the linear B‐cell epitope of the target protein. In order to increase the prediction accuracy, two servers, ABCpred (https://webs.iiitd.edu.in/raghava/abcpred/ABC_submission.html;Access:2023.01) and PEPTIDES (http://imed.med.ucm.es/Tools/antigenic.html; Access:2023.01) were used for comprehensive judgement.^24^ ABCpred is a learning algorithm based on a recurrent neural network. The test comes from the Bcipep and Swiss⁃Prot database data, and its overall prediction accuracy reaches 66%. The predicted amino acid length in the study is set to 16, and the default threshold is 0.75. PEPTIDES uses the combination of Kolaskar and Tongaonkar to determine the entire amino acid sequence, and the accuracy of this method is 75%. The two servers were used to screen candidate linear B‐cell epitopes, and afterwards, the iBCE‐EL server (which can be found at http://thegleelab.org/iBCE‐EL/iBCE.html; Access:2023.01) was used to verify these epitopes.

### 2.2 The screening of T cell epitope prediction

T cell epitope prediction tool was based on the process of polypeptide fragment binding to major histocompatibility complex, intracellular processing and transport.^25^ The tool calculates a prediction score for each polypeptide as a potential T cell epitope using this information. Leukocyte antigen (LA), also known as MHC, is divided into two classes – class I and class II – which respectively activate cytotoxic T lymphocytes (CTL) and helper T lymphocytes (HTL).

In order to increase the prediction accuracy, two servers, NetMHCpan EL4.1 (http://tools.iedb.org/mhci/; Access: 2023.01) and NetMHCpan4.1 (https://services.healthtech.dtu.dk/service.php? NetMHCpan‐4.1; Access: 2023.01) were used for comprehensive judgement of CTL candidate epitope prediction.^26^, ^27^ Among them, NetMHCpan EL4.1 was based on the consensus recommendation method, and NetMHCpan4.1 was based on the artificial neural network algorithm, which evaluates the binding ability of each peptide to a specific MHC⁃class I molecule and the epitope prediction score. In this study, the pig allele threshold was close to 0, the epitope length was set to 8–14, and the top 1% of the scores were used as potential peptide vaccine candidate epitopes. Then, the immunogenicity of the candidate epitopes was investigated using the server MHC⁃I Immunogenicity (http://tools.iedb.org/immunogenicity/; Access: 2023.01) in the IEDB database, and epitopes with an immunogenicity score >0.2 were selected as the final prediction results.

HTL candidate epitope prediction was used by the server NetMHCIIpanv4.0 EL (http://tools.iedb.org/mhcii/; Access: 2023.01). NetMHCIIpanv4.0 EL used artificial neural networks to predict peptide binding to any MHCII molecule of a known sequence.^28^ The length of the predicted epitope was 15, and the screening criteria for the candidate epitope was set to the top 10% of the percentile. Based on NetMHCIIpanv4.0 EL prediction, the server IFNepitope (https://webs.iiitd.edu.in/raghava/ifnepitope/index.php; Access: 2023.01) was used to predict the induction ability of candidate epitopes to IFN⁃γ. Predicting the induction ability of candidate epitopes to IL‐4 was performed using the server IL4pred (https://webs.iiitd.edu.in/raghava/il4pred/predict.php; Access: 2023.01). The active epitopes that activated these two cytokines were further screened.

### 2.3 The design and evaluation of the candidate vaccine

Based on the epitopes obtained from the immunoinformatics as mentioned above analysis, three linkers, KK, AAY and GPGPG, were used to link the B cell, HTL and CTL epitopes together in a certain order. To improve the immunogenicity of the candidate vaccine, the 50S ribosomal protein L7/L1 molecular adjuvant was connected to the N‐terminus through the EAAAK flexible link. We use the server VaxiJen v2.0 (http://www.ddg‐pharmfac.net/vaxijen/VaxiJen/VaxiJen.html; Access:2023.02) to predict the immunogenicity of the constructed candidate vaccine. ANTIGENpro (http://scratch.proteomics.ics.uci.edu/; Access:2023.02) was used to predict the immune antigenicity of the constructed candidate vaccine, and SOLpro (http://scratch.proteomics.ics.uci.edu/index.html; Access:2023.02) was used to predict the solubility of constructed vaccine candidates. Allergenicity of the constructed candidate vaccine was using AllerTop (http://www.ddg‐pharmfac.net/AllerTOP/; Access:2023.02). The ExPASy tool (https://web.expasy.org/protparam/; Access:2023.02) was used to predict the constructed candidate vaccine's molecular weight, isoelectric point, half‐life, stability, etc.

### 2.4 Prediction of secondary and tertiary structures and disulfide bond design of candidate vaccines

Prediction of the secondary structure of candidate vaccines was made using the servers SOPMA (http://npsa‐prabi.ibcp.fr/cgi‐bin/npsa_automat.pl?page=npsa_sopma.html; Access:2023.02) and PSIPRED 4.0 (http://bioinf.cs.ucl.ac.uk/psipred; Access:2023.02). The preliminary prediction of the candidate vaccine's tertiary structure was completed using rRosetta (https://yanglab.nankai.edu.cn/trRosetta/; Access:2023.02), whose results include Omega, Phi, Distance, Theta, Contact parameters and TM‐score. To obtain a more refined model quality, the coarse structure of the model was optimized using the server Galaxy WEB (https://galaxy.seoklab.org/cgi‐bin/submit.cgi? type = REFINE; Access:2023.02). The server PROCHECK (https://saves.mbi.ucla.edu/; Access:2023.02) was used to generate Ramachandran graphs for comparing and evaluating the model quality before and after optimization. It was necessary to improve the stability of the model before proceeding with the next step. The model was designed for disulfide bonds using the server Disulfide by Design 2.0 (http://cptweb.cpt.wayne.edu/DbD2/index.php; Access:2023.02). Finally, the candidate vaccine's tertiary structure model was tested globally through the server ProSA web (https://prosa.services.came.sbg.ac.at/prosa.php; Access:2023.02).

### 2.5 Conformational epitope prediction of B cell and protein molecular docking

The refined model tertiary structure of Conformational B‐cell epitopes was predicted using the server ElliPro (http://tools.iedb.org/ellipro/; Access:2023.02). Therefore, removing water molecules and other impurities was performed using pyMol. Predicting the interactions between TLR‐3 (PDB ID: 2A0Z) as a receptor and the candidate vaccine proteins as a ligand was made using the server pyDockWEB (https://life.bsc.es/pid/pydockweb; Access:2023.02). The computer software LigPLot+ was used to determine the interface between the vaccine ligand and TLR‐3 receptor and whether there are hydrogen bonds and hydrophobic interaction between amino acid residues.^29^, ^30^

### 2.6 Immune simulation analysis and computer cloning

The decision to use immune simulation analysis was made according to classical immunology principles. To evaluate the immunogenicity and immune response pattern, the CImmSim server (available at https://150.146.2.1/C‐IMMSIM/index.php; Access: 2023.02) was employed. This server employs machine learning models to predict how different immune responses interact. According to the typical immunization process, the injections were immunized for a total of 3 times at 1‐week intervals. All simulation parameters were set to default values, and time steps were set to 1, 20 and 40 (each time step was 8 h, with time step 1 being the time point of the first immunization). Codon optimization and reverse translation were performed using the server JCat (http://www.jcat.de/; Access:2023.02), and candidate vaccine cDNA sequences were generated. Results are composed of GC content and Codon Adaptation Index (CAI) scores, which can assess protein expression levels. In addition, the optimized candidate vaccine cDNA sequence was inserted into the prokaryotic expression vector of the pET‐32a(+) vector by SnapGene computer software to ensure its stable expression in *Escherichia coli* K‐12.

## 3 RESULTS

### 3.1 Candidate epitopes of T and B lymphocytes of PRRSV GP3 and GP5 proteins

Eleven and 7 linear B‐cell epitopes of PRRSV strain GP3 and GP5 were predicted using PEPTIDES, while 11 and 9 linear B‐cell epitopes were predicted using ABCpred. Compared to the results of the two prediction methods, the epitopes containing common segments were verified with the iBCE⁃EL server. Finally, 5 and 7 polypeptide fragments were obtained as candidate B‐cell epitopes for GP3 and GP5 proteins, respectively, with the results presented in Table 1.

**TABLE 1:** Candidate B‐cell epitopes.

Protein	Name	Sequences	Start position	End position
GP3	B1	YTAQFHPEI	118	126
	B2	GIGNVS	128	133
	B3	QTYYQHQ	166	172
	B4	KPTPPQHQTS	217	226
	B5	LRRFAKVLS	242	250
GP5	B6	TACCCSRLLFLWCIVP	7	22
	B7	FDWAVET	60	66
	B8	IVSYGALTTSHFLDTV	76	91
	B9	TVSTAGYYHGRYVLSS	95	110
	B10	SWRYSCTR	133	140
	B11	RWRSPVIV	154	161
	B12	AATPLTRVSA	185	−194

One hundred and forty‐two and 104 CTL epitopes of PRRSV strain GP3 protein and 92 and 72 CTL epitopes of GP5 protein were predicted, respectively, using NetMHCpan4.1 EL and NetMHCpan4.1. After eliminating redundant and nested peptides, the immunogenicity assessment was performed using the MHC⁃I Immunogenicity tool in the IEDB database. Finally, 3 and 3 polypeptide fragments were obtained as candidate CTL epitopes for GP3, and GP5 proteins, respectively, with the results presented in Table 2.

**TABLE 2:** Candidate CTL cell epitopes.

Protein	Name	Sequences	Allele	Start position	Immunogenicity
GP3	CTL1	SENDHDELGFM	SLA‐2*0601	83	0.25738
	CTL2	GGNWFHLEW	SLA‐3‐YC SLA‐1*0401 SLA‐2‐YDL02	175	0.41438
	CTL3	VSVRVFRTL	SLA‐3*0401 SLA‐6*0103	208	0.28224
GP5	CTL4	KFDWAVETF	SLA‐1*0201 SLA‐1‐YDL01	59	0.41423
	CTL5	YRWRSPVIVEK	SLA‐3*0304 SLA‐3*0701	153	0.2109
	CTL6	KVEVEGHLI	SLA‐2*0501 SLA‐1‐YTH	166	0.22426

NetMHCIIpanv4.0 EL predicted 114 GP3 and 169 GP5 protein epitopes of PRRSV strains. After eliminating redundant and nested peptides, the servers IFNepitope and IL4pred were used to screen further the epitopes that activate IFN‐γ and IL‐4 as candidate epitopes. Then, 2 and 3 polypeptide fragments were obtained from GP3 and GP5 proteins as candidate HTL epitopes, respectively, and the results are presented in Table 3.

**TABLE 3:** Candidate HTL cell epitopes.

Protein	Name	Sequences	Allele	Start position
GP3	Th1	ISAVFQTYYQHQVDG	HLA‐DRB1*13:01; HLA‐DRB1*16:01; HLA‐DPA1*01:03/DPB1*74:01; HLA‐DRB1*08:01; HLA‐DPA1*01:03/DPB1*106:01	161
	Th2	WFLRRSPASHVSVRV	HLA‐DPA1*01:04/DPB1*20:01; HLA‐DPA1*01:03/DPB1*20:01; HLA‐DPA1*01:03/DPB1*09:01	198
GP5	Th3	VPFYLAVLANASNSN	HLA‐DRB1*04:26; HLA‐DRB1*07:17; HLA‐DQA1*01:03/DQB1*04:05	21
	Th‐4	WRYSCTRYTNFLLDT	HLA‐DPA1*03:03/DPB1*90:01; HLA‐DPA1*01:03/DPB1*65:01	134
	Th‐5	RSPVIVEKGGKVEVE	HLA‐DRB1*03:04; HLA‐DRB5*01:05; HLA‐DPA1*01: 04/DPB1*04:02; HLA‐DRB1*03:15; HLA‐DPA1*01:06/DPB1*53:01; HLA‐DQA1*01:01/DQB1*02:04	156

### 3.2 Construction of the candidate vaccines

**FIGURE 1:**
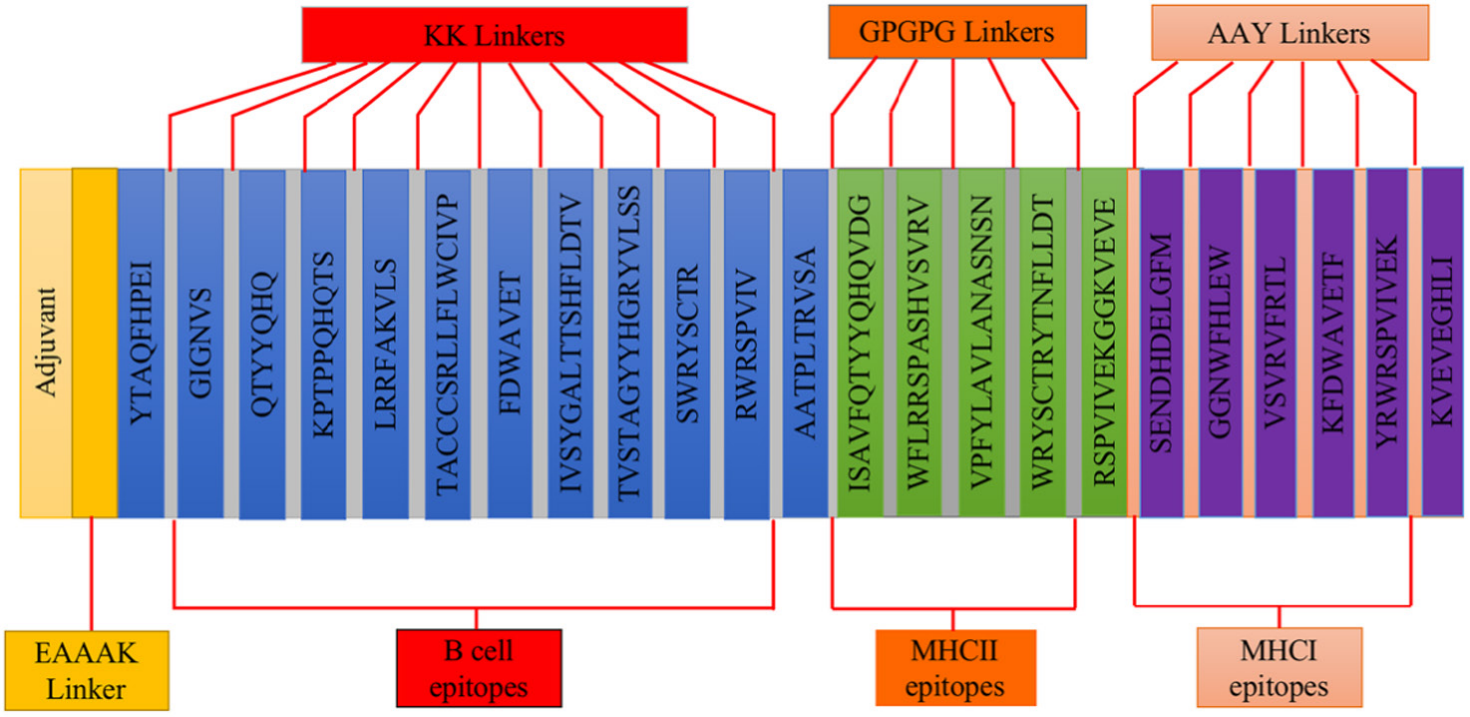
The schematic diagram of candidate vaccine construction.

Using the candidate epitopes screened through immunoinformatics, linkers KK, AAY and GPGPG were used to link candidate B cell, HTL and CTL epitopes together in a certain order. 50S ribosomal protein L7/L1 molecules act as immunopotentiators and promote long‐lasting immune responses by linking to the N‐terminus of the construct using an EAAAK linker. This reduces linkages to other protein regions and improves stability through efficient separation. At the same time, it maintains its independent immunogenic activity by using a double lysine (KK) linker. Using AAY and GGPPG linkers can enhance the recognition ability of vaccine subunits. A linker can ensure good stability, domain orientation and folding rate. Therefore, the constructed vaccine's amino acid sequence is:

MAKLSTDELLDAFKEMTLLELSDFVKKFEETFEVTAAAPVAVAAAGAAPAGAAVEAAEEQSEFDVILEAAGDKKIGVIKVVREIVSGLGLKEAKDLVDGAPKPLLEKVAKEAADEAKAKLEAAGATVTVKEAAAKYTAQFHPEIKKGIGNVSKKQTYYQHQKKKPTPPQHQTSKKLRRFAKVLSKKTACCCSRLLFLWCIVPKKFDWAVETKKIVSYGALTTSHFLDTVKKTVSTAGYYHGRYVLSSKKSWRYSCTRKKRWRSPVIVKKAATPLTRVSAGPGPGISAVFQTYYQHQVDGGPGPGWFLRRSPASHVSVRVGPGPGVPFYLAVLANASNSNGPGPGWRYSCTRYTNFLLDTGPGPGRSPVIVEKGGKVEVEAAYSENDHDELGFMAAYGGNWFHLEWAAYVSVRVFRTLAAYKFDWAVETFAAYYRWRSPVIVEKAAYKVEVEGHLI. A schematic diagram of candidate vaccine construction is shown in Figure 1.

### 3.3 The physicochemical property analysis and secondary structure prediction of the candidate vaccine evaluation

**FIGURE 2:**
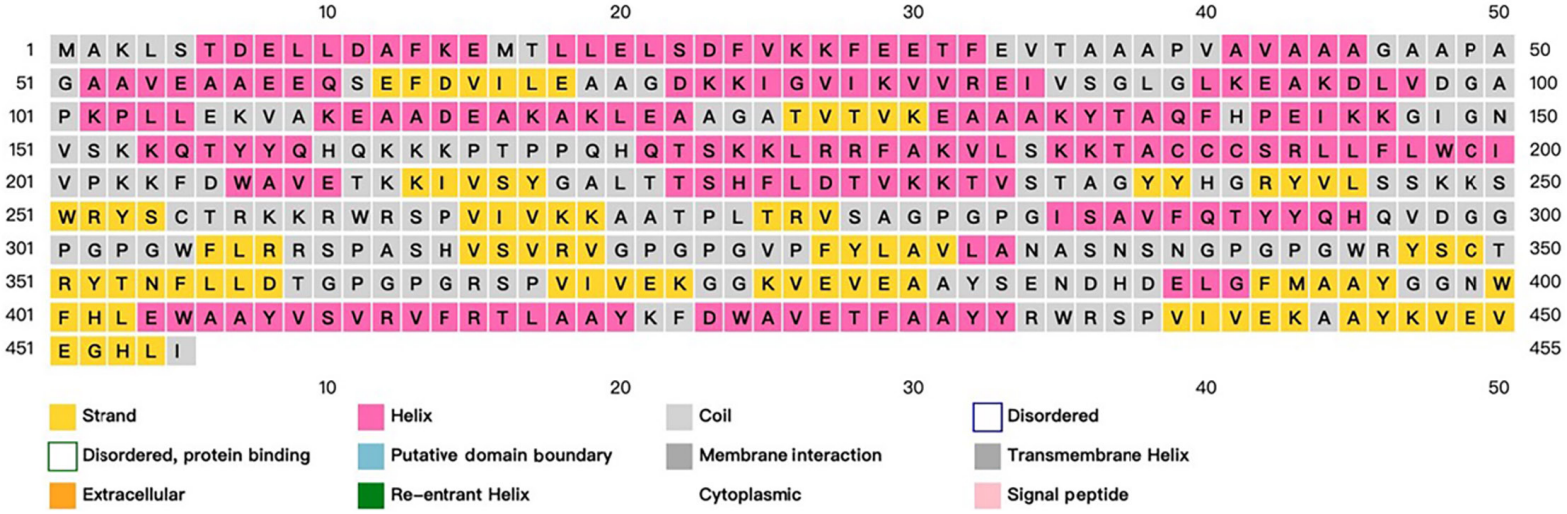
The prediction and analysis of the secondary structure of candidate vaccines. Different colours represent corresponding information about the secondary structure of a protein.

Vaxijenv2.0 predicted the protective antigen, and when the threshold was set at 0.4, the candidate vaccine score was 0.5137, suggesting it could be used as an antigen. Antigen immunogenicity predicted by ANTIGENpro was 0.783261, suggesting that the candidate vaccine had good immunogenicity. SOLpro predicted that the candidate vaccine overexpression solubility was 0.783261, suggesting that it is soluble. AllerTOP V2.0 predicts the allergenicity of candidate vaccines, and the results show that the candidate vaccines were non‐allergenic proteins. ExPASy calculates that the candidate vaccine has a full length of 455 amino acids, a molecular weight of 49.85033 kD, and an isoelectric point of 9.46. The antigen has a half‐life of 30 h in vitro in mammalian reticulocytes, more than 20 h in vivo in yeast cells and more than 10 h in *Escherichia coli*. The antigen stability index was also measured to be 31.87, and the prediction results classify it as a stable protein. Grand average of hydropathicity (GRAVY): −0.230 represents the average value of protein sequence hydrophobicity. The aliphatic index was 78.51, representing the protein's thermal stability, and the higher aliphatic index of the protein results in better thermal stability. The secondary structure of candidate vaccines was predicted by PSIPRED and SOPMA, showing that candidate vaccines were mainly composed of Alpha helix: 39.12%, Extended strand: 21.76%, Beta turn: 9.67% and Random coil: 29.45%. The results of secondary structure prediction are shown in Figure 2.

### 3.4 Modelling, refinement and evaluation of the tertiary structure of candidate vaccines

**FIGURE 3:**
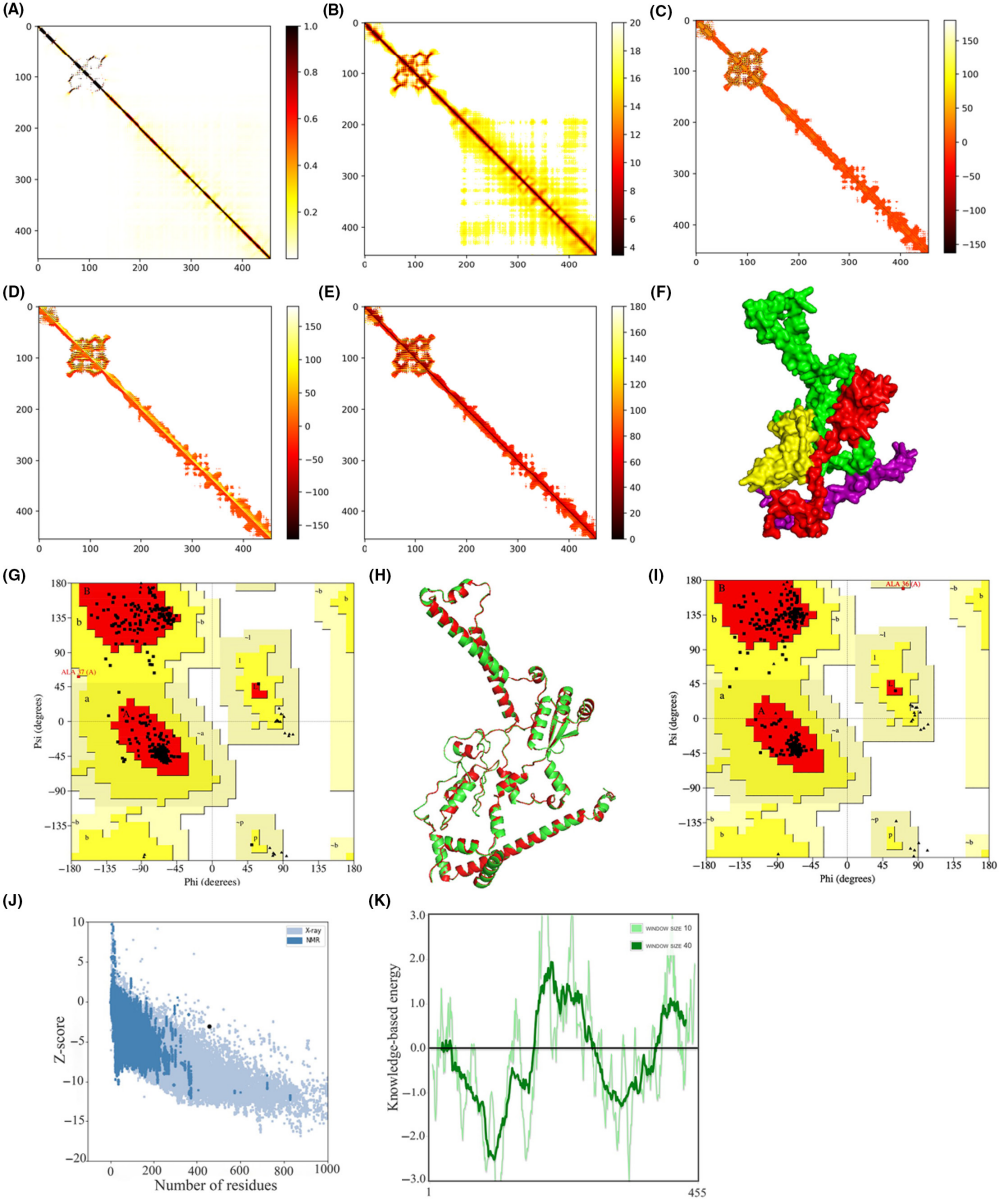
Modelling, refinement and evaluation of the tertiary structure of candidate vaccines. (A) The Omega parameter of the two‐dimensional structure; (B) The Phi parameter of the two‐dimensional structure; (C) The Distance parameter of the two‐dimensional structure; (D) The Theta parameter of the two‐dimensional structure; (E) The Contact parameter of the two‐dimensional structure; (F) Within the tertiary structure of the candidate vaccine, the 50S ribosomal L7/L12 molecular adjuvant is coloured in red, while the B‐cell epitope is green, the HTL epitope is yellow, and the CTL epitope is purple. The TM‐score of the structure is 0.358; (G) To verify the rough model using Ramachandran drawing; (H) The structure comparison between the rough model and the refined model, the rough model and the fine model are green and red, respectively; (I) To verify the refined model using Ramachandran drawing; (J) The *Z* score of the refined model ProSA SEB map is −3.03; (K) Local model quality assessment.

The tertiary structure of the candidate vaccine was modelled and generated by server trRosettaJ, and the result is shown in Figure 3F. The server synchronously generated the 2D visualization views (contact diagram, distance diagram and direction diagram), including Contact, Distance, omega, theta and phi, and the results are shown in Figure 3A–E. Further, the Ramachandran diagrams were generated using the server EBI PDBSum to evaluate the quality of the rough model; the results are shown in Figure 3G. The results show that the rough model most favoured regions (A, B, L) is 95.4%, additional allowed regions (a, b, l, p) is 4.3%, generously allowed regions (~a, ~b, ~l, ~p) is 0.3%, and disallowed regions is 0.0%. Galaxy‐Refine is a server that enhances and improves a given coarse model structure. In this case, the server could generate five refined models, with the top model—referred to as the number one model—having a Rama‐favoured score of 98.9%. This score is the highest among all of the refined models, making it the best option. The computer software Pymol was used to draw a cartoon comparison map, and the result is shown in Figure 3H. The refined model structure was verified through Ramachandran drawing, and the result is shown in Figure 3I. The results show that the refined model most favoured regions (A, B, L) is 97.5%, additional allowed regions (a, b, l, p) is 2.3%, generously allowed regions (~a, ~b, ~l, ~p) is 0.0%, and disallowed regions is 0.3%. Compared with the rough model, the quality of the refined model of GalaxyRefine was improved. In order to improve the stability of the protein and study the interaction between proteins, the server Disulfide by Design2.0 was further used for disulfide design. The prediction results show that the B‐factors are all 0, indicating that the candidate vaccine protein modelling was stable enough and did not require further disulfide bond design. Finally, the final model structure was scored and validated using the server ProSA web. The structural precision analysis revealed a *z* value of −3.33, which falls within the acceptable range for natural proteins. The results of this analysis are displayed in Figure 3J. The local model quality's energy score is moderate, as depicted in Figure 3K. As a result, the refined model can be used for further analysis.

### 3.5 The analysis and molecular docking of candidate vaccine and conformational B‐cell epitope

**FIGURE 4:**
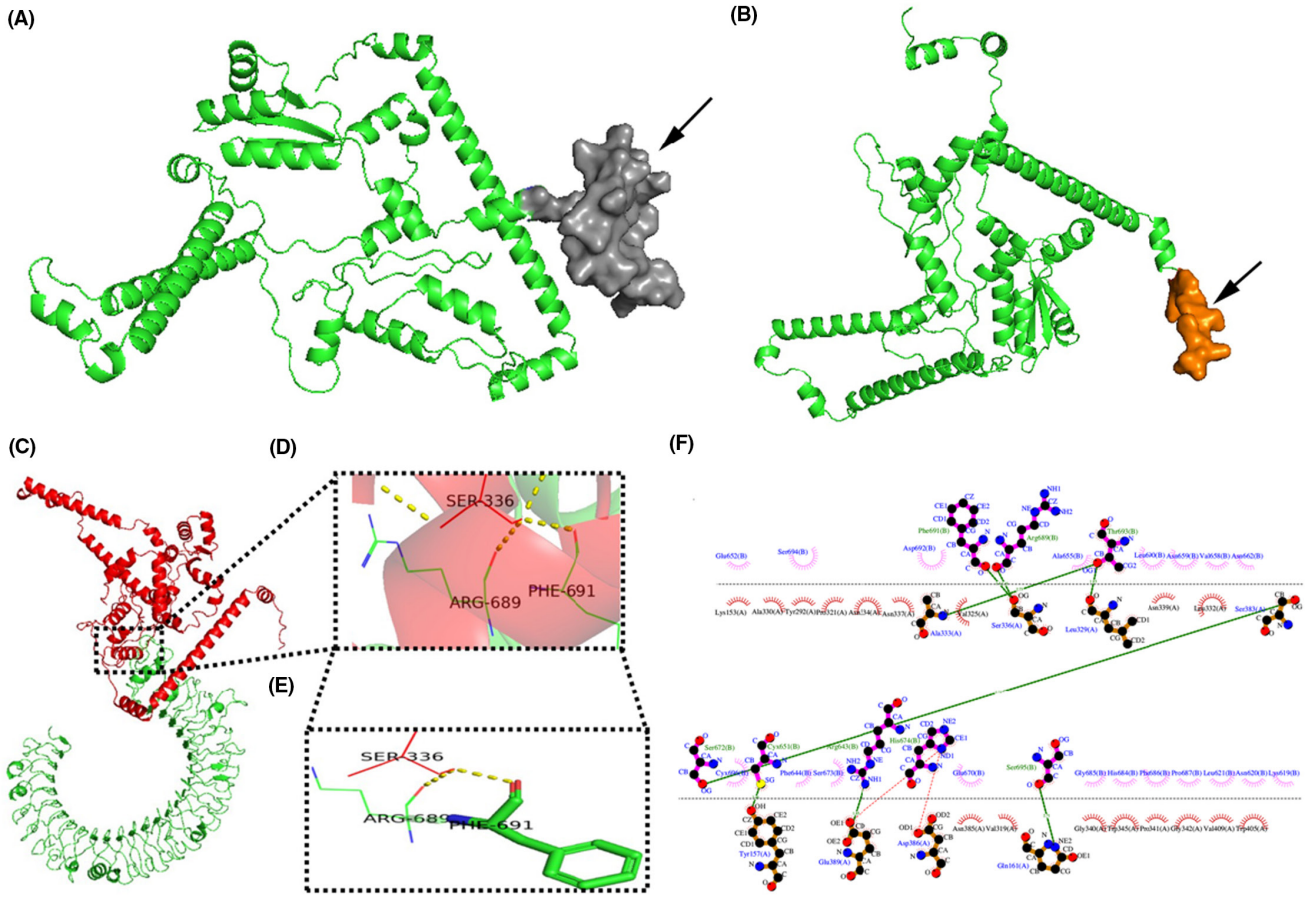
B cell‐dominant epitope map and candidates Docking results of vaccines and TLR3 molecules. (A) The highest‐scoring linear epitope binding position; (B) the highest‐scoring discontinuous epitope‐binding position. (C) The candidate vaccine structure is shown in red and the TLR3 structure in green; (D) Partial zoom‐in shows amino acid residues; (E) PDBsum draws a two‐dimensional interaction diagram; (F) Schematic diagram of the interaction between protein chains.

The server ElliPro was used to predict 7 linear and 8 discontinuous epitopes based on the refined tertiary structure model of the candidate vaccines. The analysis revealed that the linear epitope binding position with the highest score was 0.818, indicated by the arrow in Figure 4A. Meanwhile, the highest score for the discontinuous epitope binding position was 0.873, shown by the arrow in Figure 4B. Both scores exceeded the set threshold of 0.5 by a significant margin. The results confirm that the designed Multi‐epitope vaccines possess superior immunogenicity. The server PyDockWEB performed rigid docking of the protein and candidate vaccine ligand with the TLR‐3 receptor protein to further evaluate their potential. This process generated 102 models, with model 24 ranking first among all prediction models. Specific values associated with this model include Conf (2753), Electrostatics (−37.883), Desolvation (−38.572), VdW (68.406) and Total (−69.614), which are shown in Figure 4C. The candidate vaccine was observed to bind deeply in the centre of TLR‐3, and specific amino acid residues were amplified locally. These observations are depicted in Figure 4D,E. To determine the amino acid residues with hydrogen bonds between the candidate vaccine ligand and TLR‐3 receptor, LigPlot+ software was used to generate a two‐dimensional interaction interface. The residues with hydrogen bonds were Phe691, Arg689, Ala655, Thr693, Cys651, Arg643, His674 and Ser695. The amino acid residues with hydrophobic interactions are: Glu652, Ser694, Asp692, Ala655, Leu690, Asn659, Val658, Asn662, Cyx696, Phe644, Ser673, Glu670, Gly685, His684, Phe686, Pro687, Leu621, Asn620 and Lys619 (Figure 4F).

### 3.6 In silico stimulation of immune responses

**FIGURE 5:**
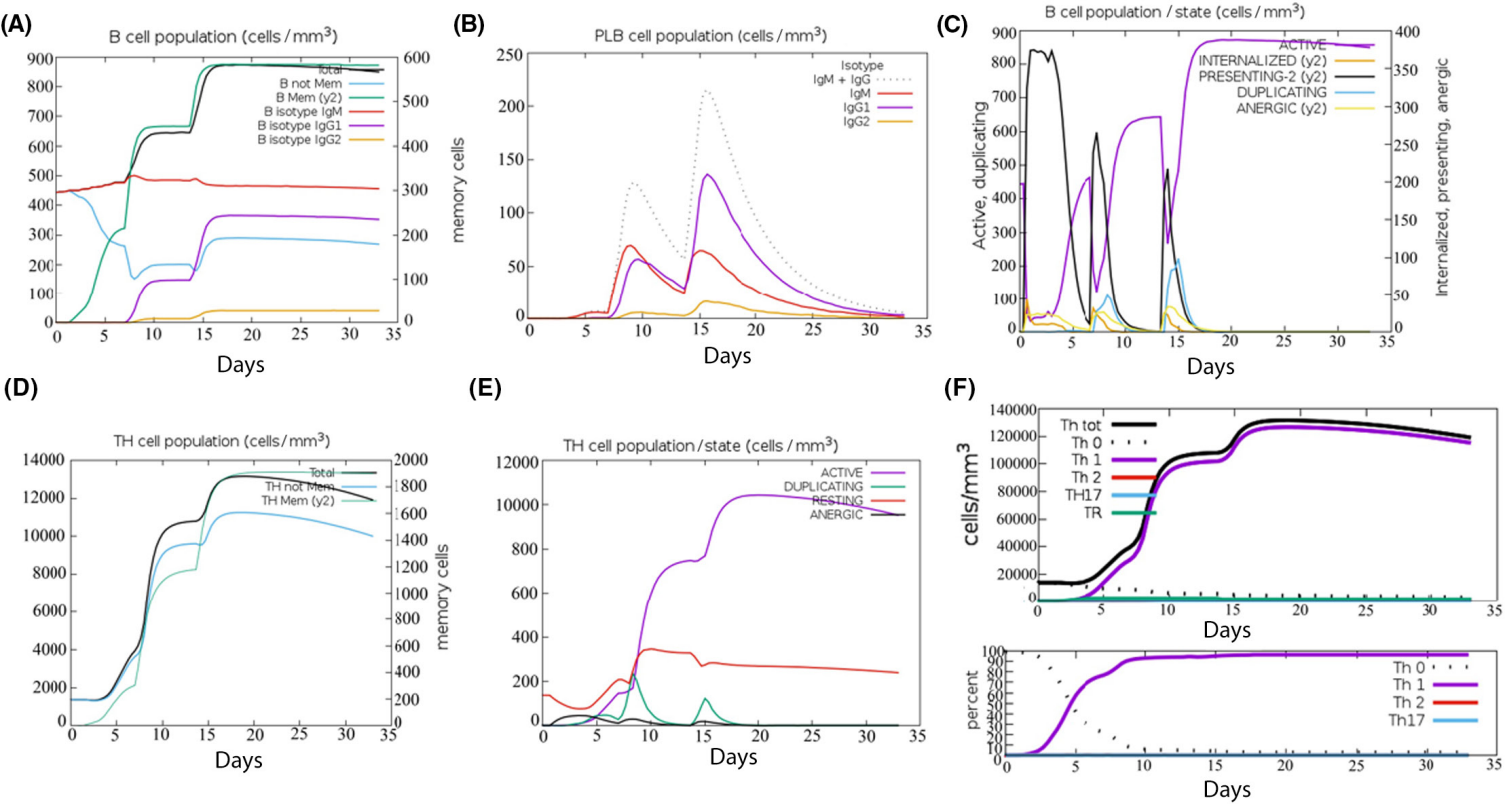
The changes in the number of immune B cells and Th in simulated immune stimulation. (A) The total counts of B‐lymphocyte, memory cells and subtypes IgM, IgG1 and IgG2; (B) The plasma B‐lymphocyte count broken down by isotype (IgM, IgG1 and IgG2); (C) B‐lymphocyte populations per entity status; (D) the counts of CD4 helper T lymphocytes. The figure shows total and memory counts; (E) CD4 helper T lymphocyte counts per entity status; (F) the counts of CD4 T‐regulatory lymphocytes.

**FIGURE 6:**
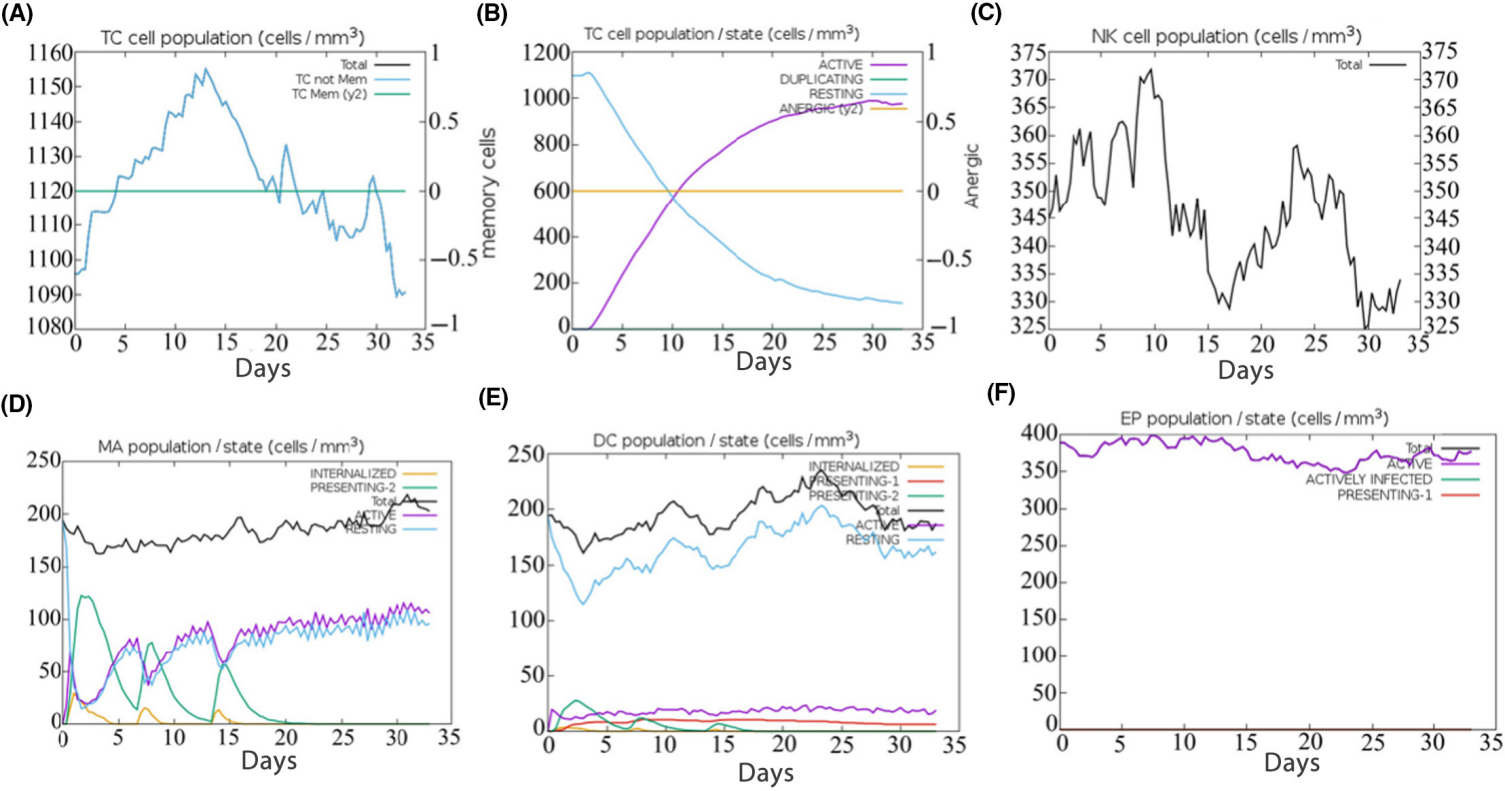
The number changes of immune CTL, NK, MA, DC and EP cells after simulated immune stimulation. (A) The counts of CD8 T cytotoxic lymphocytes. Total and memory are shown; (B) the counts of CD8 T cytotoxic lymphocyte per entity state; (C) the counts of natural killer cells; (D) the counts of internalized and presented macrophage; (E) Dendritic cells can present antigenic peptides on MHC class I and II molecules; (F) the total counts of epithelial cells can be disaggregated into active, viral infection and presentation on MHC class I molecules.

**FIGURE 7:**
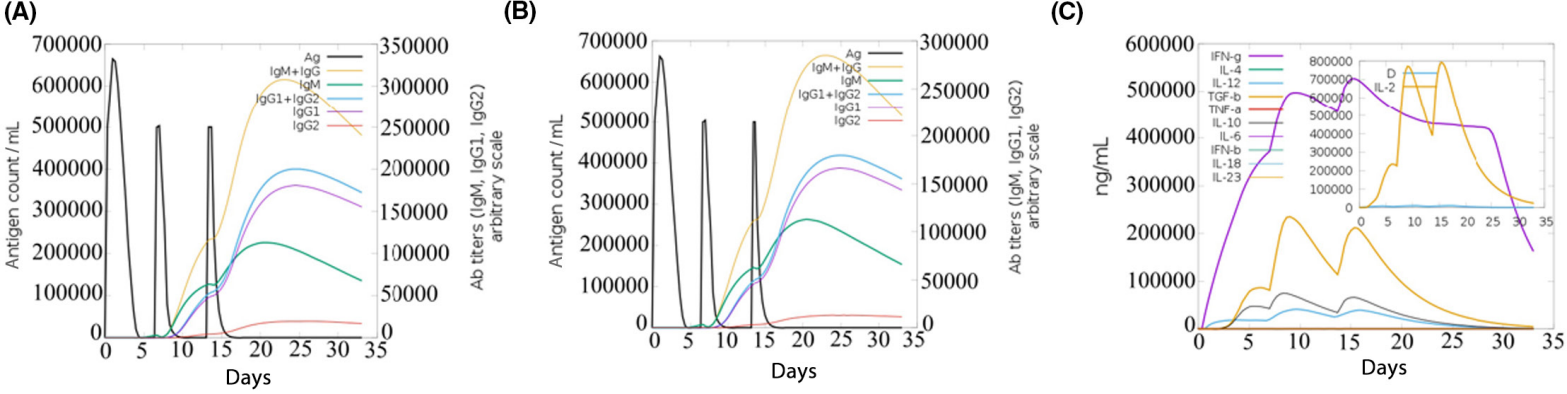
Trends of immunoglobulins and immune complexes and concentration trends of cytokines and interleukins. (A) Antigens and immunoglobulins. Antibodies were subdivided by isotype (with 50S ribosomal L7/L12 molecular adjuvant); (B) antigen and immunoglobulin. Antibodies were subdivided by isotype (without 50S ribosomal L7/L12 molecular adjuvants); (C) the concentrations of cytokines and interleukins.

The C‐ImmSim server can evaluate candidate vaccine immunogenicity using computer simulation, enabling the rapid screening of candidates. The simulation results demonstrated that B cells exhibited high activity within the first 5 days after three stimulations, leading to significant increases in the B‐cell population and antibody levels of IgG1 + IgG2, IgM and IgG + IgM. TH and TC cells activated exponentially within the same time frame, stimulating memory cells. The pattern of cytokines released showed a high secretion of IFN‐γ, followed by IL‐2, indicating high levels of activation within 0–15 days before reaching saturation. The vaccine significantly activated macrophages and natural killer cells, while the numbers of dendritic and epithelial cells remained at approximately 200 cells/mm^3^ and 350–400 cells/mm^3^, respectively. The simulation results demonstrated a high induction level, generating a strong immune response, as shown in Figures 5, 6, 7. According to C‐ImmSim's simulation results, the candidate vaccine conforms to the law of inducing an immune response, exhibits good immunogenicity and effectively activates humoral and cellular immunity.

### 3.7 Codon optimization and in silico cloning

**FIGURE 8:**
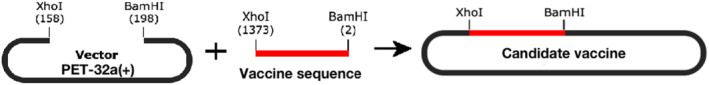
Schematic diagram of the connection between candidate vaccine and pET‐32a(+) prokaryotic expression vector.

The pET‐32a(+) prokaryotic expression vector was selected using computer simulation cloning. The reverse translation of the candidate vaccine's amino acid sequence was completed using the server JCat, which obtained the expression gene sequence of the candidate vaccine. A better expression can be obtained by codon optimization. The optimized sequence had a CAI improvement value of 1 and a GC content of 49.89%, within the optimal range of 30%–70%. Thus, the candidate vaccine is suitable for expression in Escherichia coli. The prokaryotic expression plasmid was constructed using BamHI and XhoI restriction endonuclease sites, as depicted in Figure 8.

## 4 DISCUSSION

In the Medium and Long‐Term Animal Disease Prevention and Control Plan (2012–2020),^31^ HP‐PRRS is one of the five first‐class animal diseases needing priority prevention and control. Currently, HP‐PRRS is an infectious disease that poses a huge threat to the development of the pig industry worldwide.^32^ Therefore, it is of great practical significance and value to do a good job of preventing and controlling HP‐PRRSV. However, few commercial vaccines are available in the market, and most are inactivated or attenuated vaccines administered by injection.^33^, ^34^ It is usually fully protective against PRRSV homologous to vaccine antigens but only partially protective against heterologous PRRSV infection.^35^ At present, the poor immunogenicity and the limited cross‐protection effect are important problems in the immunology research of PRRS vaccines.^36^ However, using live attenuated vaccines raises concerns about virulence reversal or genetic recombination with field strains.^37^ PRRSV is the virus most prone to genetic recombination and replacement among all RNA viruses. In addition, the inactivated vaccines cannot effectively mediate cellular immunity. Therefore, developing and applying new vaccines may bring opportunities for the precise prevention and control of the disease.

Currently, relevant research results show that using GP3 and GP5 proteins as exogenous vaccine target proteins can produce better immune effects.^38^ Based on this conclusion, the study intends to analyse, screen and design a candidate vaccine that fuses the dominant protective epitopes of GP3 and GP5 proteins in tandem through immunoinformatics. This method differs from traditional vaccine antigen epitopes and is simpler, faster and safer. Multiple B‐ or T‐lymphocyte epitopes on this candidate vaccine virus protein are integrated into specific vectors. The constructed candidate vaccine can effectively stimulate the body to trigger humoral and cellular immunity and enhance the host's protective immune response, which helps control infection.

The study found that B‐cell epitopes play a role in inducing antibody production and mediating their potent characteristics. CTLs limit pathogen spread by secreting unique antiviral cytokines which recognize and destroy infected cells.^39^ HTL epitopes are paired with more alleles for greater population coverage.^40^ Therefore, in this study, we applied the amino acid sequences of GP3 and GP5 proteins to predict and screen B cell, HTL and CTL epitopes. After the dominant epitope and molecular adjuvant were connected in series through a flexible linker, a candidate vaccine was constructed. It underwent evaluation using bioinformatics software to ensure the candidate vaccine was safe. The software checked for several qualities, including high antigenicity and immunogenicity, as well as non‐allergic and non‐toxic properties. Additionally, the software assessed the vaccine's solubility and stability. These data suggest the vaccine candidate can elicit a strong immune response without an allergic reaction. This study performed correlation analysis simultaneously at the proteins' secondary and tertiary structure levels. The results show that in the secondary structure, the candidate vaccine β‐turn and random coil structure account for a large proportion, which is more prone to torsion. The structure protrudes outwards and mostly appears on the surface of protein antigens, stimulating the body to produce an immune response. The tertiary structure prediction provides extensive knowledge of protein spatial arrangement, which supports studying ligand interaction and protein function. After improvement, most of the residues of the candidate vaccine structure exist in the allowed area, and there are very few residues in the unallowed area. In the docking with the immune receptor molecule TLR‐3, 8 amino acid residues with hydrogen bonds and 19 amino acid residues with hydrophobic interactions were observed. Its docking binding requires less energy, suggesting that the candidate vaccine can induce an innate immune response. The analysis of the C‐IMMSIM immune stimulation test found that the immunoglobulin was significantly increased to a large extent. In addition, a significant increase in the levels of active T cytotoxicity and T helper lymphocytes was observed, which enhanced cellular and adaptive immune responses. IFN‐γ also remained at peak levels during the injection period. This indicates that the candidate vaccine can generate an immune response. Furthermore, it can induce CD8 + T cells and generate CD4 + T helper type 1 cell immune response, causing humoral and cellular immune responses, indicating good antiviral properties. Finally, the candidate vaccine was codon‐optimized and cloned into a prokaryotic expression vector, showing high‐level expression of the candidate vaccine in bacteria. However, whether the candidate vaccine constructed in this study can stimulate B and T cells remains to be experimentally verified. The candidate vaccines need to be tested in vitro and in vivo, which should be of considerable value in immune responses to candidate anti‐PRRSV vaccines in future studies.

## 5 CONCLUSION

This study successfully predicted and screened the dominant epitopes of PRRSV GP3 and GP5 proteins using immunoinformatics. The flexible linker is connected in series with B‐cell epitopes, CTL and HTL epitopes to construct candidate vaccines using molecular adjuvants. The candidate vaccines, including comprehensive secondary structure analysis, tertiary structure modelling and optimization, molecular docking, codon optimization, reverse translation and computer cloning, were evaluated. The constructed candidate vaccine may provide a relevant theoretical basis and data support for the prevention, control and vaccine development for PRRSV.

## AUTHOR CONTRIBUTIONS

**Dongyu Liu:** Data curation (equal); funding acquisition (equal); software (equal); validation (equal). **Yaping Chen:** Conceptualization (equal); data curation (equal); formal analysis (equal); funding acquisition (equal); methodology (equal); writing – original draft (equal); writing – review and editing (equal).

## CONFLICT OF INTEREST STATEMENT

The authors declare no conflict of interest.

## Data Availability

Data openly available in a public repository that issues datasets with DOIs.
